# A Rare Case of Moderate Pericardial Effusion Uncovering Severe Hypothyroidism

**DOI:** 10.7759/cureus.84566

**Published:** 2025-05-21

**Authors:** Hasan Ilyas, Muhammad Haider, Caio Furtado, Amrit Gautam, Touqir Zahra

**Affiliations:** 1 Department of Internal Medicine, Florida Atlantic University Charles E. Schmidt College of Medicine, Boca Raton, USA

**Keywords:** cellulitis, hypothyroidism, hypothyroid pericardial effusion, levothyroxine, pericardial effusion, pericardial tamponade

## Abstract

Severe hypothyroidism can manifest with various cardiovascular complications, including bradycardia, diastolic dysfunction, and, less commonly, pericardial effusion, due to thyroid hormone’s regulatory effects on cardiac function. While small pericardial effusions may be seen in clinical practice, large effusions and tamponade are rare. We present a case of a 64-year-old female patient with obesity who was initially admitted for bilateral lower extremity cellulitis. During her hospitalization, clinical signs such as lethargy, puffy facies, and slow speech raised concern for hypothyroidism, prompting thyroid function testing, which revealed severe hypothyroidism (thyroid-stimulating hormone 58.3 mIU/L, free T4 0.51 ng/dL). A transthoracic echocardiogram, obtained as part of further evaluation, demonstrated a moderate circumferential pericardial effusion without tamponade physiology. She was managed conservatively with oral levothyroxine, and cardiology advised against pericardiocentesis due to hemodynamic stability. Pericardial effusion in hypothyroidism typically resolves with hormone-replacement therapy. This case underscores the importance of considering hypothyroidism in patients with nonspecific systemic symptoms and highlights its potential to cause clinically significant but reversible cardiovascular complications.

## Introduction

Thyroid hormones regulate cardiovascular function primarily through T3, influencing heart rate, contractility, vascular resistance, and lipid metabolism. In hypothyroidism, these effects are disrupted, leading to bradycardia, diastolic dysfunction, hyperlipidemia, and pericardial effusion [1]. The effusion is attributed to increased capillary permeability, albumin leakage, and mucopolysaccharide accumulation in the pericardium [2]. While large pericardial effusions may develop, tamponade is rare due to the slow accumulation and adaptive stretch of the pericardium. Echocardiography plays a critical role in detecting such effusions and guiding management. The incidence of pericardial effusion varies with the severity of hypothyroidism, ranging from 3% to 6% in mild cases to nearly 80% in advanced stages such as myxedema coma [3].

Clinically, pericardial effusion in hypothyroidism is often asymptomatic, though severe cases can progress to pericardial tamponade, presenting with hemodynamic instability. Early identification and management of hypothyroidism-induced pericardial effusion are crucial to preventing complications. Treatment primarily involves thyroid hormone replacement with levothyroxine (LTX), which typically resolves pericardial effusion as thyroid function normalizes. In rare cases of significant hemodynamic compromise, pericardiocentesis may be required.

This case highlights the diagnostic challenges and clinical significance of hypothyroidism-induced pericardial effusion in a patient who presented with bilateral lower extremity cellulitis and nonspecific systemic symptoms. In this patient, initial concern centered around an infectious or inflammatory process, such as systemic sepsis or connective tissue disease. However, the absence of fever, leukocytosis, or autoimmune markers made systemic lupus or vasculitis less likely. Additionally, malignancy-related effusion was considered, but the lack of constitutional symptoms and imaging findings made this unlikely. The presence of classic hypothyroid features and confirmatory lab findings redirected the diagnostic focus. This case underscores the importance of considering endocrine etiologies such as hypothyroidism in patients with nonspecific presentations. Recognizing such patterns will allow clinicians to be aware of potentially serious but reversible causes of systemic illness.

## Case presentation

A 64-year-old female patient with a past medical history of obesity (BMI 31) and hypertension presented to the emergency department at Delray Medical Center on July 21, 2024, with bilateral lower extremity swelling, erythema, and a large ulcer on the right lower leg, accompanied by a draining wound on the left leg. She reported fatigue and constipation and appeared disheveled and withdrawn. Per collateral history, she had not sought medical care in over 40 years and had missed work due to ongoing lethargy.

Vital signs on admission were unremarkable except blood pressure of 169/100 mmHg. On physical examination, she appeared fatigued, with puffy facies, loss of outer eyebrows, dry skin, slow speech, and bilateral lower extremity edema and ulcers. No jugular venous distension or audible pericardial rub was noted.

Differential diagnosis considered for her bilateral lower extremity edema and ulcers included heart failure, lymphedema, and cellulitis. She was initially treated for presumed bilateral lower extremity cellulitis and started empirically on intravenous vancomycin and piperacillin-tazobactam. Wound cultures later revealed heavy growth of *Morganella morganii*, *Enterococcus faecalis*, and *Pseudomonas aeruginosa*, suggesting a polymicrobial infection of moderate to severe intensity, likely due to chronic wound exposure and impaired skin barrier. The patient underwent surgical incision and drainage on hospital day 2.

**Figure 1:**
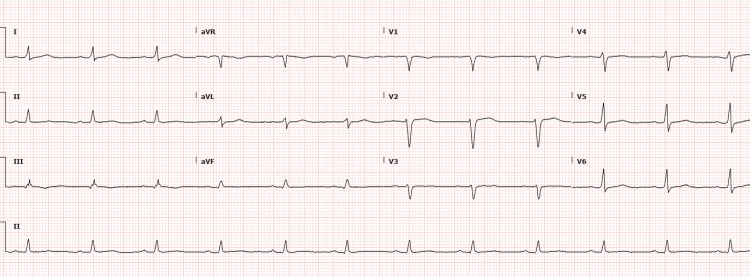
EKG showing normal sinus rhythm and low-voltage QRS EKG: electrocardiogram; aVR: augmented vector right; aVL: augmented vector left; aVF: augmented vector foot

Despite initial antimicrobial therapy, the patient’s profound fatigue, slow mentation, and physical findings prompted broader diagnostic evaluation. Thyroid function tests drawn within 48 hours of admission revealed a thyroid-stimulating hormone (TSH) of 58.3 µIU/mL and free T4 of 0.51 ng/dL, consistent with severe primary hypothyroidism. She denied any known history of thyroid disease but endorsed chronic fatigue, hair thinning, and longstanding constipation. An electrocardiogram (EKG) showed a normal sinus rhythm and low-voltage QRS complexes, as shown in Figure 1.

**Figure 2:**
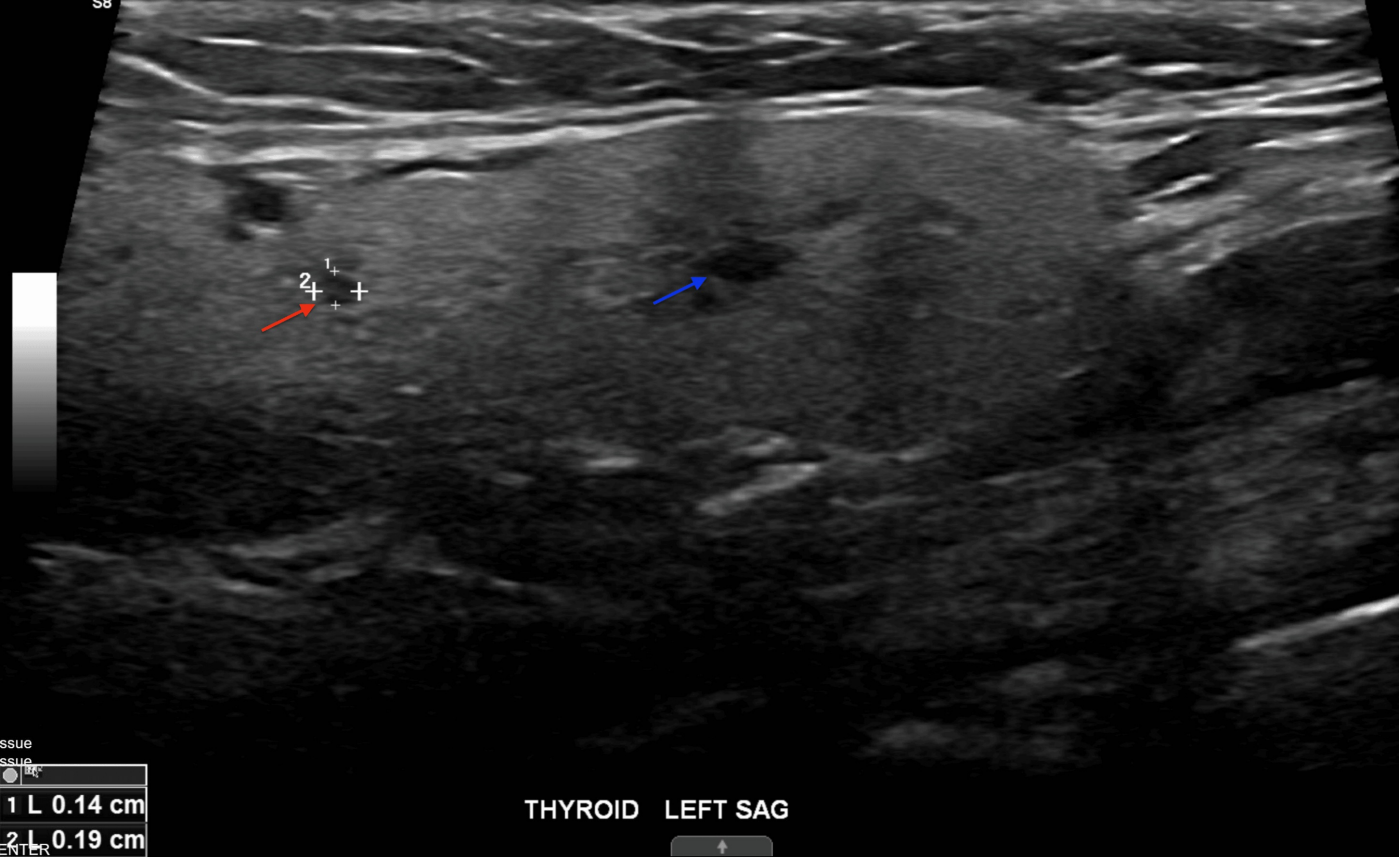
A simple cyst in the left lobe of the thyroid gland (blue and red arrows)

A thyroid ultrasound was also performed to assess structural abnormalities such as goiter, thyroiditis, or mass. It showed a normal-sized, slightly heterogeneous thyroid gland with two simple cysts in the left lobe (3 and 4 mm), considered incidental findings, as shown in Figure 2.

**Figure 3:**
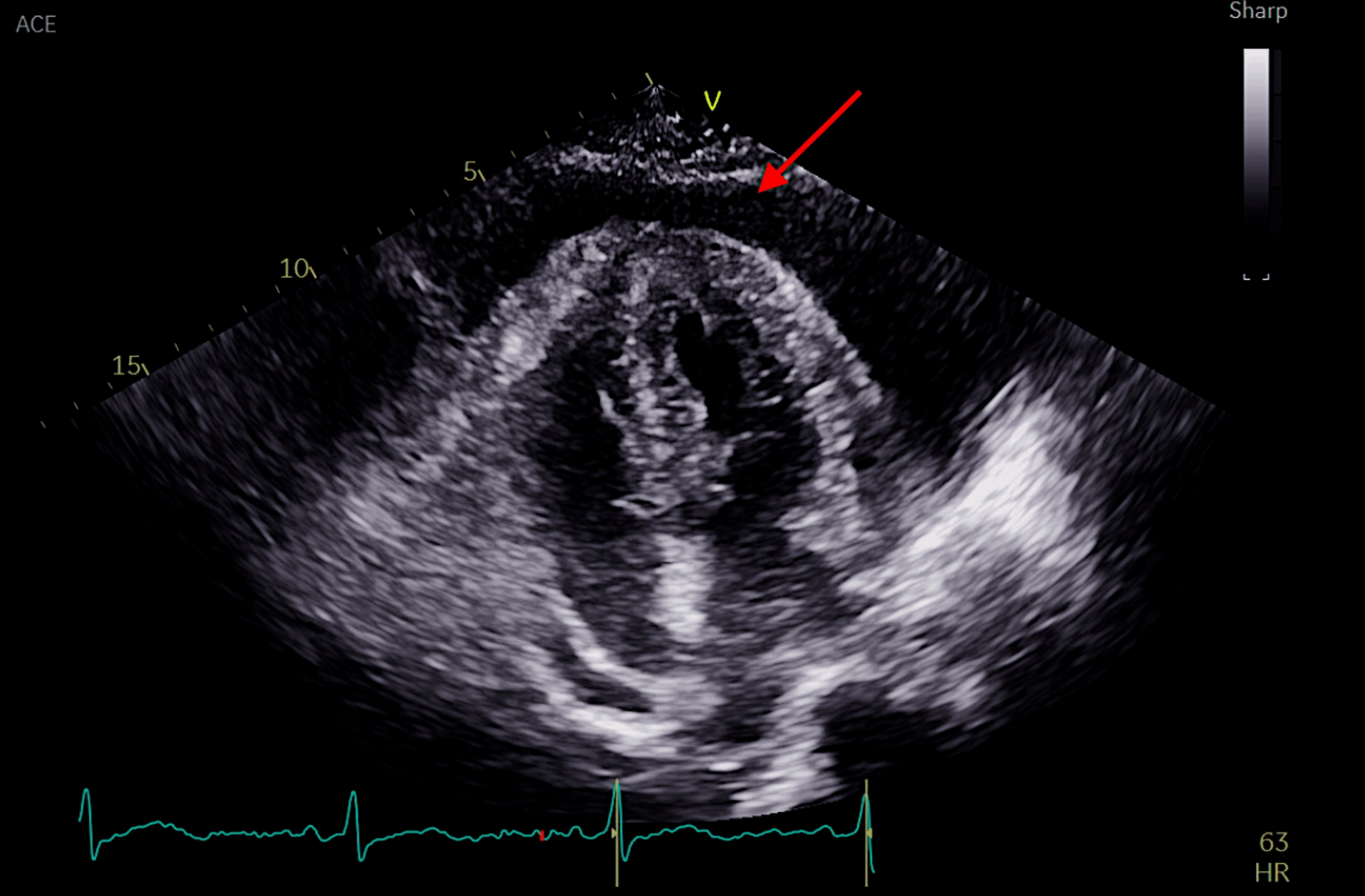
Apical four-chamber view on TTE depicting pericardial effusion (red arrow) TTE: transthoracic echocardiogram

Given the EKG findings and systemic signs, including lower extremity edema, a transthoracic echocardiogram (TTE) was obtained on hospital day 5 to evaluate for cardiac abnormalities. The TTE revealed a moderate circumferential pericardial effusion with pericardial thickening and minimal right atrial (RA) compression, but no evidence of right ventricular collapse or tamponade physiology, as depicted in Figure 3. The effusion was interpreted as chronic in the setting of longstanding hypothyroidism, and conservative management was recommended.

Lower extremity arterial Doppler studies were performed due to concern for underlying peripheral arterial disease contributing to poor wound healing. These confirmed severe arterial insufficiency. Vascular surgery was consulted, revascularization was not pursued during hospitalization, but risk factor modification with statins and antiplatelet therapy was initiated.

Endocrinology was consulted, and the patient was started on oral LTX 150 mcg daily based on clinical severity and estimated weight-based dosing. Although this dose may be higher than typical starting doses for older adults, it was selected due to the absence of active cardiac ischemia and the need to address profound hormone deficiency. A plan was made for close TSH and free T4 monitoring postdischarge, with outpatient endocrinology follow-up for dose titration.

Repeat TTE was not performed during the hospital stay as the patient remained hemodynamically stable, and conservative management was deemed appropriate. Outpatient echocardiographic follow-up was arranged to assess resolution of the effusion over time. The patient was discharged in stable condition with follow-up appointments in endocrinology, cardiology, infectious disease, and vascular surgery.

## Discussion

The incidence of pericardial effusion in hypothyroidism varies by severity, ranging from 3% to 6% in mild cases to up to 80% in myxedema coma [3]. The term “myxedema heart,” first described in 1918 [4], refers to a constellation of cardiac manifestations seen in profound hypothyroidism, including bradycardia, low voltage on EKG, reduced myocardial contractility, and, in some cases, pericardial effusion. Our patient’s presentation, characterized by low-voltage QRS on EKG, diminished energy, and echocardiographic findings of large effusion, was consistent with this entity.

Pericardial effusion in hypothyroidism results from increased capillary permeability, impaired lymphatic drainage, and sodium and water retention. In this case, these mechanisms likely contributed to the large, slowly accumulating effusion. The minimal RA compression seen on TTE further supports the chronic nature of the effusion, as hypothyroid-related effusions tend to accumulate gradually, allowing the pericardium to stretch and avoid overt tamponade physiology.

TTE was prompted by a combination of nonspecific systemic features (lethargy, puffy facies, and slowed speech), in addition to low-voltage QRS on EKG, a classic but often overlooked clue suggestive of pericardial effusion. Low-voltage QRS complexes are seen on an EKG due to the insulating effects of fluid around the heart. Physical examination did not reveal signs of tamponade such as pulsus paradoxus, elevated jugular venous pressure (JVP), or muffled heart sounds, which further supported the decision to image rather than intervene urgently.

While TTE remains the first-line modality for evaluating pericardial effusion and cardiac function, adjunctive imaging such as cardiac CT or MRI can be particularly useful when the etiology is unclear or to assess pericardial thickening, chronicity, and potential constriction. These modalities provide better tissue characterization and can help rule out malignancy, tuberculosis, or connective tissue disease-related effusions in ambiguous cases.

Management of pericardial effusion in hypothyroidism is typically conservative in the absence of tamponade. In our case, clinical criteria guiding the decision to avoid pericardiocentesis included hemodynamic stability, lack of hypotension or tachycardia, and echocardiographic absence of right ventricular diastolic collapse or significant chamber compression. Conversely, pericardiocentesis would be indicated if signs of tamponade develop, such as hypotension, elevated JVP, tachycardia, pulsus paradoxus, or sonographic evidence of right-sided chamber collapse [5]. Our approach was in line with previously reported cases by Dinleyici et al. [6] and Wang et al. [3], where hypothyroidism-associated pericardial effusion was successfully managed conservatively with thyroid hormone replacement, without the need for pericardiocentesis in hemodynamically stable patients.

Pericardial effusions related to hypothyroidism generally begin to resolve within two to four weeks of LTX initiation, with full resolution typically occurring over several weeks to a few months, depending on the degree of hypothyroidism and the volume of effusion [7]. Follow-up imaging is usually performed at four- to six-week intervals or earlier if new symptoms arise (e.g., worsening dyspnea, chest discomfort, or signs of hemodynamic compromise).

Diuretics should be avoided in large effusions, especially in hemodynamically borderline patients. Diuretics reduce intravascular volume, which can precipitate chamber collapse and lead to acute decompensation in effusions that were previously well tolerated due to pericardial compliance. Case reports have documented tamponade precipitated by aggressive diuresis in similar patients [8].

This case reinforces the importance of considering hypothyroidism in patients with nonspecific systemic complaints and shows how integrating clinical suspicion, targeted imaging, and judicious management can prevent unnecessary interventions and promote recovery.

## Conclusions

This is a valuable learning case due to its atypical presentation: a presumed infectious process ultimately led to the diagnosis of severe hypothyroidism with a large pericardial effusion, remarkable for its absence of tamponade physiology. It highlights the diagnostic value of systemic symptoms such as lethargy, puffy facies, and slowed speech in prompting evaluation beyond the initial working diagnosis. The multidisciplinary care was essential: infectious disease tailored antibiotics for polymicrobial cellulitis, vascular surgery addressed peripheral arterial disease and risk factor management, endocrinology initiated thyroid hormone replacement, and cardiology guided conservative effusion management.

Follow-up typically includes repeat transthoracic echocardiography every four to six weeks and thyroid function monitoring. With adequate hormone replacement, most effusions resolve over weeks to months. While recurrence is rare with sustained euthyroidism, patients should be educated about warning signs such as fatigue, dyspnea, or leg swelling, especially if nonadherence or delayed resolution is a concern. This case underscores the importance of considering endocrine etiologies in nonspecific presentations and the role of integrated care in preventing complications, however our case is limited by the absence of long-term follow-up data.
